# Epitope Mapping of Human Polyclonal Antibodies to the fHbp Antigen of a *Neisseria Meningitidis* Vaccine by Hydrogen-Deuterium Exchange Mass Spectrometry (HDX-MS)

**DOI:** 10.1016/j.mcpro.2024.100734

**Published:** 2024-02-09

**Authors:** Laura R. Grauslund, Susanne Ständer, Daniele Veggi, Emanuele Andreano, Kasper D. Rand, Nathalie Norais

**Affiliations:** 1 Protein Analysis Group, Department of Pharmacy, University of Copenhagen, Copenhagen, Denmark; 2 GSK Vaccines, GSK, Siena, Italy; 3 Monoclonal Antibody Discovery (MAD) Lab, Fondazione Toscana Life Sciences, Siena, Italy

**Keywords:** HDX-MS, epitope mapping, human polyclonal antibody, fHbp, MenB, 4CMenB

## Abstract

Antigen-antibody interactions play a key role in the immune response post vaccination and the mechanism of action of antibody-based biopharmaceuticals. 4CMenB is a multicomponent vaccine against *Neisseria meningitidis* serogroup B in which factor H binding protein (fHbp) is one of the key antigens. In this study, we use hydrogen/deuterium exchange mass spectrometry (HDX-MS) to identify epitopes in fHbp recognized by polyclonal antibodies (pAb) from two human donors (HDs) vaccinated with 4CMenB. Our HDX-MS data reveal several epitopes recognized by the complex mixture of human pAb. Furthermore, we show that the pAb from the two HDs recognize the same epitope regions. Epitope mapping of total pAb and purified fHbp-specific pAb from the same HD reveals that the two antibody samples recognize the same main epitopes, showing that HDX-MS based epitope mapping can, in this case at least, be performed directly using total IgG pAb samples that have not undergone Ab-selective purification. Two monoclonal antibodies (mAb) were previously produced from B-cell repertoire sequences from one of the HDs and used for epitope mapping of fHbp with HDX-MS. The epitopes identified for the pAb from the same HD in this study, overlap with the epitopes recognized by the two individual mAbs. Overall, HDX-MS epitope mapping appears highly suitable for simultaneous identification of epitopes recognized by pAb from human donors and to thus both guide vaccine development and study basic human immunity to pathogens, including viruses.

## Highlights

• HDX-MS can identify epitopes of human polyclonal (pAb) antibodies.
• HDX-MS allows epitope mapping of pAb samples without Ab-selective purification.
• Epitope mapping of pAb from donors vaccinated against *Neisseria meningitidis* (MenB).
• Identification of immuno-dominant pAb epitopes on fHbp antigen from the 4CMenB vaccine.
• HDX-MS provides unique information for vaccine design and studying human immunity.

## Graphical Abstract

**Figure:**
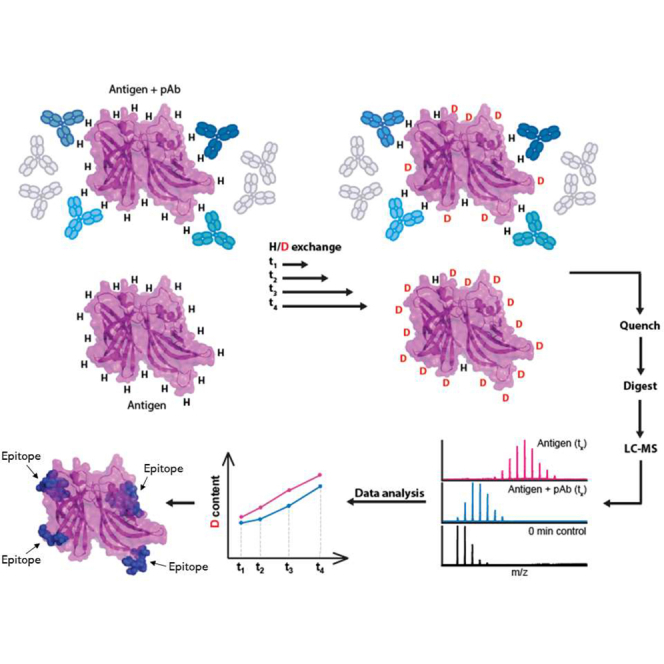

## In Brief

Antigen-antibody interactions play a key role in the immune response post-vaccination. In this study, we use hydrogen/deuterium exchange mass spectrometry (HDX-MS) to identify epitopes in an antigen recognized by polyclonal antibodies (pAb) from two vaccinated human donors (HDs). Our HDX-MS data reveal several epitopes recognized by the complex mixture of human pAb in both HDs with high similarity. Furthermore, we show that the main epitopes can be identified without the need for antigen-specific enrichment of the pAb samples.

Antigen–antibody interactions are central to pathogen immunity. Antibodies induce immune responses through binding to linear or conformational epitopes on their target antigen. Identification of these epitopes is crucial for the fundamental understanding of immune responses as well as for development of therapeutic antibodies and efficient vaccine antigens. Various biochemical techniques can be used to identify linear or conformational epitopes, like protein chip, etc (1, 2). However, especially identification of conformational or discontinuous epitopes can be challenging with such routine biochemical methods although it is known that structure can be important for immunogenicity (3). Hydrogen-deuterium exchange mass spectrometry (HDX-MS) is a sensitive analytical technique to probe the impact of binding between proteins and has therefore emerged as a useful and very sensitive technique for epitope mapping of conformational epitopes (4). Most HDX-MS epitope mapping studies are focused on the complex formed by an antigen and a monoclonal antibody (mAb) or the antigen-binding fragment (Fab) (5, 6). Such studies reveal detailed structural information about the epitopes recognized by individual mAbs, but require isolation of B cells, gene sequencing and recombinant production and purification of each antibody of interest which is a time-consuming process. The B cells used for sequencing are most often isolated from peripheral blood (PB), which is estimated to account for only 2% of the total population of B cells (7, 8). The number of plasma cells, the antibody-secreting B cells, in PB depends on the immune status of the individual (9). Studies have shown that 1 week after vaccination, the antigen-specific plasma cells constitute around 30% of the total population of plasma cells in PB (10, 11). Furthermore, out of the 100 serum IgG clonotypes identified, three of the clonotypes were present in such high concentrations that they comprised almost half of the total serum IgG population (11). The complexity of a human serum polyclonal antibody population (pAb) is thus dependent on the immune status of the individual and the time after infection which means that the number of antigen-specific antibodies in the pAb sample will vary. Thus, little is known about how well the mAbs sequenced from PB represent the serum antibodies present after infection or vaccination. In addition, individual antibodies present in a serum pAb sample against a given antigen will bind with different binding affinities and may be antagonistic. Vice versa, it has been shown that antibodies without neutralizing or bactericidal activity alone can obtain synergistic activity effects as pairs or as oligoclonal mixtures (12, 13, 14, 15). Thus, the epitopes recognized by individual mAbs sequenced from PB-derived B cells cannot be assumed to be additive and directly reflect the collective epitopes recognized by the full Ab population in a pAb sample.

HDX-MS has proved to be useful for epitope mapping with antibody samples of increasing complexity. One study has used different combinations of up to four human mAbs (16). Other studies have shown the feasibility of using antigen-specific pAb purified/enriched from immunized goats (17) or mice (18). To our knowledge, HDX-MS has so far not been used for epitope mapping of human pAb samples without enrichment of antigen-specific pAb (19).

Here, we use an optimized HDX-MS approach to map the binding of both unpurified and enriched human pAb samples to the factor H binding protein (fHbp) (20). fHbp is one of the main antigens in the 4CMenB vaccine against *Neisseria meningitidis* serogroup B (MenB) (21). In MenB, fHbp is a surface-exposed lipoprotein that plays a key role in evading human immune responses through binding to human complement factor H (22). The C-terminal of fHbp consists of an eight-stranded antiparallel β-barrel while the N-terminal consists of an antiparallel β-sheet and two small helical segments with high intrinsic flexibility (23). Using HDX-MS, we successfully map the collective epitopes on fHbp of pAb populations isolated from two human donors (HDs) vaccinated with 4CMenB. Our data shows that the pAb from both HDs impact similar regions of fHbp providing important insights into the immunodominant regions on fHbp that are recognized by the human immune response. From a methodological perspective, our results highlight how HDX-MS can be used to identify the coexisting epitopes that are collectively recognized by distinct human pAb populations.

## Experimental Procedures

### Enrollment of Bexsero Vaccinees for Human Sample Collection

Human samples from healthy adults immunized with multicomponent serogroup B meningococcal vaccines containing recombinant fHbp variant 1, were obtained from a Phase I clinical study conducted in Krakow, Poland, and sponsored by 10.13039/100004336Novartis Vaccine, now part of the GSK group of Companies and from an observational unblinded and not randomized clinical trial conducted in Siena, Italy, and sponsored by Fondazione Toscana Life Sciences. The Clinical trial protocols were approved by the Bioethics Committee of the District Medical Doctors’ Chamber in Krakow and the Comitato Etico di Area Vasta Sud Est ethics committees, respectively. Studies were conducted according to good clinical practice in accordance with the Declaration of Helsinki (European Council 2001, US Code of Federal Regulations, ICH 1997). Written informed consents were obtained from the subjects.

### Purification of Polyclonal and fHbp-Specific Antibodies

Antibodies were purified from two 4CMenB-vaccinated human individuals, here referred to as HD1 and HD2. Serum from HD1 was collected 30 days after administration of the third dose of the vaccine, 3 months after administration of the first dose. Serum from HD2 was collected 30 days after administration of the first dose of the vaccine. Extraction of polyclonal antibodies and the subsequent purification of fHbp-specific antibodies were performed using a peristaltic pump or an ÄKTA purifier (GE Healthcare) using the same workflow. Before purification, serum was filtered through a sterile filter (Millex GP 0.22 μm) and diluted 1:1 (v/v) with binding buffer (300 mM NaCl, 50 mM NaP, pH 7.2). Purification of polyclonal antibodies was performed using two connected HiTrap 5 ml HP columns (GE Healthcare) containing Protein A and G, respectively. For further purification of fHbp-specific antibodies, 10 mg recombinant fHbp was immobilized on a 5 ml NHS HP SpinTrap using the corresponding buffer kit (GE Healthcare) according to the manufacturer’s protocol. The samples were loaded on the columns at a flow rate of 3 ml/min until complete loading of the total sample volume. After complete loading of the sample, the column was washed with 50 ml binding buffer at a flow rate of 5 ml/min. The antibodies were eluted from the column at a flow rate of 5 ml/min with 25 ml elution buffer (100 mM glycine, pH 2.6) into 0.5 ml fractions collected in tubes containing neutralizing buffer (1 M Tris, pH 9.0). Fractions were run on a NuPAGE four to 12% Bis-Tris gel (Invitrogen) and fractions containing antibody were pooled and dialyzed (PBS, pH 7.4) at 5°C overnight.

### Hydrogen-Deuterium Exchange

HDX-MS analysis was performed using the following samples: a) fHbp in absence or presence of pAb (HD1) in Ag:Ab ratios 1:2, 1:6 or 1:12 (7.5 pmol fHbp and 15 pmol, 45 pmol, or 90 pmol pAb, respectively), was diluted in 99.9% D_2_O (PBS, pH 7.4) to a final D_2_O content of 90% and quenched after 10 min, 100 min and 24 h; b) fHbp in absence or presence of pAb (HD1) in Ag:Ab ratio 1:9 (10 pmol fHbp and 90 pmol pAb), was diluted in 99.9% D_2_O (PBS, pH 7.4) with or without 0.5 M urea to a final D_2_O content of 90% and quenched after 15 s, 1 min, 10 min (in triplicate), 100 min and 24 h; c) fHbp in absence or presence of pAb (HD2) in Ag:Ab ratio 1:20 (7.5 pmol fHbp and 150 pmol pAb), was diluted in 99.9% D_2_O (PBS, pH 7.4) with or without 0.5 M urea or 0.5 M NaCl to a final D_2_O content of 90% and quenched after 15 s and 10 min (both in triplicates); d) fHbp in absence or presence of fHbp-specific pAb (HD2) in Ag:Ab ratio 1:2 and 1:5 (5 pmol fHbp and 10 pmol pAb or 25 pmol pAb, respectively), was diluted in 99.9% D_2_O (PBS, pH 7.4) to a final D_2_O content of 90% and quenched after 15 s, 10 min, 100 min (all in triplicates). Samples were pre-incubated at 30 min followed by deuterium labeling at 25 °C. Aliquots (50 μl) were removed from the labeling reaction at the specified time intervals and quenched 1:1 to a final pH of 2.5 in ice-cold quench buffer (4 M GndHCl, 215 mM KH_2_PO_4_, pH 2.3) and immediately frozen to −80 °C until LC-MS analysis. Maximally labeled controls were prepared in triplicate by incubating fHbp in 6M deuterated GndHCl for 24 h at 25 °C (final D_2_O content of 90%).

### HDX-MS Analyses

Quenched deuterated protein samples were quickly thawed and immediately injected onto a UPLC system (NanoACQUITY HDX technology, Waters) coupled to a hybrid Q-TOF Synapt G2-Si mass spectrometer (Waters). The UPLC system was operated at 0°C and equipped with an in-house packed pepsin column (IDEX) containing pepsin immobilized on agarose beads (Thermo Scientific Pierce), a trap C18 column (ACQUITY UPLC BEH C18 1.7 μm VanGuard column Waters)) and an analytical C18 column (ACQUITY UPLC BEH C18 1.7 μm, 1 x 100 mm column (Waters)). Online protein digestion was performed at 20°C at a flow rate of 200 μl/min with mobile phase A (0.23% (v/v) formic acid in mQ water, pH 2.5), and the generated peptic peptides were trapped and desalted for 3 min. Peptides were eluted form the trap and onto the analytical column at a flow rate of 40 μl/min and a 7 min gradient from 8% to 40% of mobile phase B (0.23% (v/v) formic acid in acetonitrile) and into the Synapt G2-Si mass spectrometer for mass analysis. The ESI source was operated in positive ion mode and the instrument was enabled for ion mobility analysis. Glu-Fibrinopeptide (Sigma-Aldrich, St Louis, USA) was used for internal calibration and as reference lock-spray signal.

For the identification of peptides, non-deuterated samples of fHbp were analyzed with the same LC method and peptides were fragmented by collision-induced dissociation (CID) using data-dependent acquisition (DDA) mode. Peptide identifications were made through the processing of MS/MS data using ProteinLynx Global Server (PLGS) version 3.0 (Waters) with a peptide tolerance of 15 ppm and a fragment tolerance of 0.1 Da. Peptides with a ladder score below four were discarded and the remaining peptides were manually evaluated through inspection of the fragmentation spectra. HDX-MS data was processed in DynamX 3.0 (Waters) and all peptides were manually checked and mass spectra with significant signal overlap or a S/N ratio below 10 were excluded from the analysis.

Back-exchange was calculated for each of the analyzed peptides, as described in the community-based recommendations for HDX-MS experiments (24). The HDX results were mapped onto the fHbp crystal structure (PDB code 3KVD) using PyMOL. To allow access to the HDX data of this study, the HDX Data Tables and the HDX Summary Tables (supplemnetal Tables S1–S10) are included in the Supporting Information according to the community-based recommendations (24).

### Experimental Design and Statistical Rationale

Samples from epitope mapping with pAb from HD1 and HD2 were analyzed as technical triplicates. To provide a robust framework for comparing the differences in HDX between two experiments, a threshold value for a statistically significant difference was calculated based on an approach modified from (25, 26) using all data points in both experiments for which triplicate measurements were available.

A statistical significance threshold was calculated based on the pooled standard deviation of all peptides in the HDX measurements performed in triplicates with a 99% confidence interval as described previously (26).

## Results

### Optimizing Experimental Conditions for HDX-MS-Based Epitope Mapping Using Human pAb

**Fig. 1:**
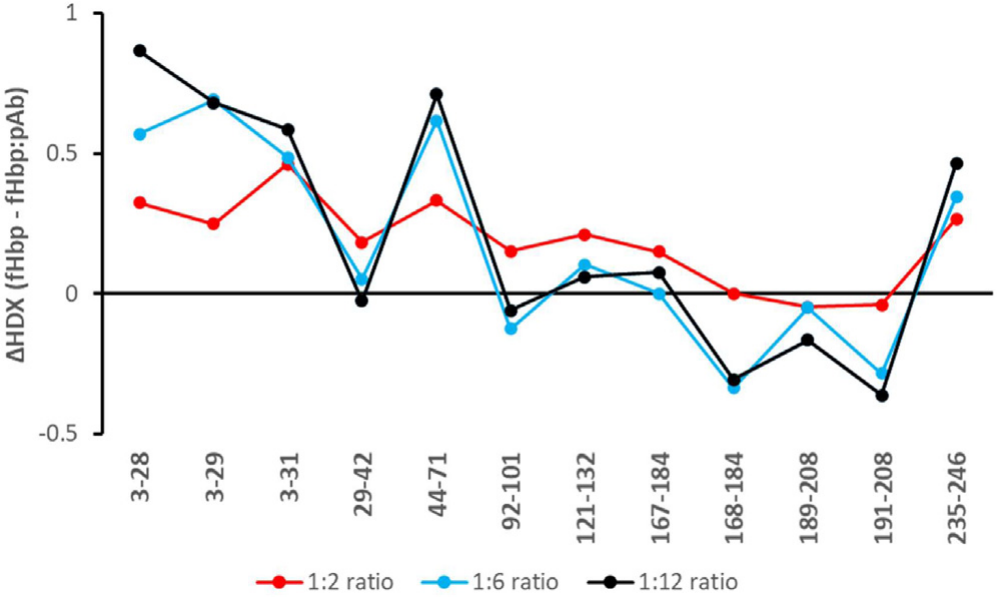
**Investigating the impact of different pAb concentrations on the HDX of fHbp**. The ΔHDX is depicted on the y-axis and the identified peptides are listed on the x-axis starting from the N-terminus of fHbp. Positive and negative ΔHDX values in the presence of pAb indicate reduced and increased HDX, respectively. ΔHDX data is shown here only for the 24 h timepoint and for the three fHbp:pAb ratio that were screened; 1:2 (*red*), 1:6 (*blue*), and 1:12 (*black*).

We extracted pAb from 4CMenB-vaccinated HD1 and performed a preliminary screening HDX-MS experiment using this sample to investigate if: i) the pAb sample contained enough fHbp-specific Ab for binding effects to be detected, and ii) an optimal ratio of fHbp:pAb required for probing binding effects could be established. In this initial HDX-MS experiment, three ratios of fHbp:pAb (1:2, 1:6 and 1:12) were used. In this preliminary experiment, only the HDX of 12 peptides could be reliably monitored across all three ratios, providing a sequence coverage of 54%. The test showed that peptides comprising residues 3 to 31, 44 to 71 and 235 to 246 were protected from HDX in presence of pAb in all three ratios (Fig. 1).

Generally, the presence of pAb in ratios 1:6 and 1:12 resulted in very similar uptake differences. In peptides 3 to 31 and 44 to 71, the 1:2 ratio showed less protection from HDX than the two other ratios, while peptides 29 to 42 and 92 to 184 showed more protection from HDX in the 1:2 ratio. Overall, the test showed first that the pAb samples do contain enough fHbp-specific Ab for binding effects to be probed. Furthermore, fHbp:pAb ratios above 1:6 provides the largest HDX differences. Although the test was performed without replicates, it indicated that peptides 3 to 31 and 44 to 71 were protected from HDX in the presence of pAb, which could suggest that those regions are potential epitopes or regions that are conformationally linked to binding.

### Epitope Mapping of fHbp With pAb From HD1

The pAb extracted from HD1 was subsequently used for a comprehensive HDX-MS epitope mapping experiment. The epitope mapping experiment was performed using a 1:9 fHbp:pAb ratio. To investigate the presence of unwanted unspecific binding effects, the HDX-MS experiment was performed both in the absence and presence of 0.5 M urea as described previously (20). A difference plot of fHbp with and without 0.5 M urea was plotted to assess any significant influence (supplemental Fig. S3). At the 10 and 100 min timepoints no significant uptake difference could be detected in any of the peptides. At the 15 s, 1 min and 24 h timepoint some peptides covering residues 3 to 31 and 44 to 71 showed small differences in HDX. Thus, 0.5 M urea had only minor impact on the HDX of two regions of fHbp and did not appear to cause major changes to the native conformation of the protein.

**Fig. 2:**
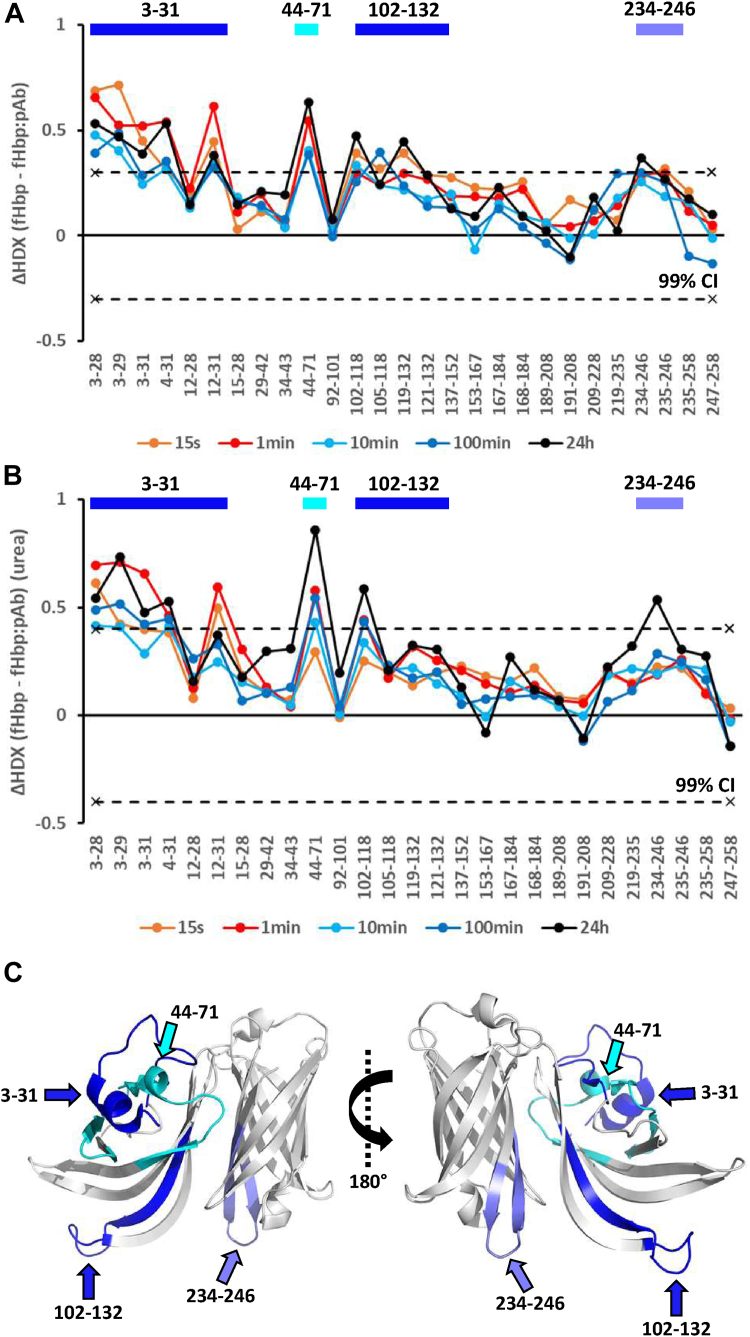
**Epitope mapping of fHbp with pAb from HD1.** The epitope mapping was done in (*A*) absence and (*B*) presence of 0.5 M urea. The ΔHDX is depicted on the y-axis and the identified peptides are listed on the x-axis starting from the N-terminus of fHbp. Positive and negative ΔHDX values in the presence of pAb indicate reduced and induced HDX, respectively. The *dotted lines* indicate the 99% CI (ΔHDX_fHbp_ ± 0.3 D and ΔHDX_urea_ ± 0.4 D). *C*, the peptides showing significant reduction in HDX in the presence of pAb are illustrated (in *blue* or *cyan* for clarity) on the crystal structure of fHbp (PDB 3KVD) using PyMOL.

The HDX of a total of 27 peptides of fHbp could be reliably monitored, corresponding to a sequence coverage of 88%. Peptides that were not covered were in most cases identified in the absence of pAb but could not be confidently identified in the presence of pAb due to spectral overlap. The impact of pAb on the HDX of fHbp was very similar in the absence and presence of urea (Fig. 2). In absence of urea, the peptides comprising residues 3 to 31, 44 to 71, and 102 to 132 showed significant decreases in HDX in the presence of pAb, while peptide 234 to 246 showed a smaller yet significant decrease in HDX in presence of pAb (Fig. 2*A*). In presence of urea, peptides comprising residues 3 to 31, 44 to 71, 102 to 118 showed significant and pronounced decreases in HDX in presence of pAb, while also peptide 234 to 246 showed a smaller yet still significant decrease in HDX (Fig. 2*B*).

Thus, the main difference observed upon the addition of urea was seen in residues 102 to 132, where peptides covering all residues showed significant protection from HDX in the absence of urea, while only residues 102 to 118 showed a significant reduction in HDX in presence of urea. The changes in HDX observed in the region of residues 119 to 132 without urea present were therefore ascribed to non-specific binding effects and was not considered to be part of the dominant epitopes recognized by the pAb from HD1. The regions with significant decrease in HDX in presence of pAb and urea form a continuous surface on fHbp, extending from one side of the bottom of the C-terminal β-barrel, across several strands of the N-terminal antiparallel β-sheet to encompass the highly dynamic N-terminal helices (Fig. 2*C*). In conclusion, the immunodominant epitopes of fHbp recognized by pAb from HD1, localize to residues 3 to 31, 44 to 71, 102 to 118, while residues 234 to 246 might comprise a less dominant epitope.

Two mAbs (1A12 and 1A3) have previously been produced from B-cell repertoire sequences from HD1 (13). The epitopes on fHbp recognized by the two mAbs have been investigated with HDX-MS (27). The epitope recognized by mAb 1A12 comprised residues 228 to 245 and the epitope recognized by mAb 1A3 comprised residues 2 to 27 and 101 to 119. The immuno-dominant epitopes of fHbp comprising residues 3 to 31 and 102 to 118 identified for HD1 pAb are thus in excellent agreement with encompassing the epitope of mAb 1A3. The residues 234 to 246 that showed small yet significant protection from HDX in presence of pAb, overlap with the epitope recognized by mAb 1A12. The epitope comprising residues 44 to 71 is not recognized by any of the two mAbs and has not been previously reported for any mAb. This highlights the importance of performing epitope mapping experiments also with pAb samples and could indicate either that the pAb sample has a high concentration of a few high affinity mAbs that are responsible for the main humoral response, or that the pAb sample consists of many different mAbs that recognize the same immunodominant mAbs.

### Epitope Mapping of fHbp With pAb From HD2.

**Fig. 3:**
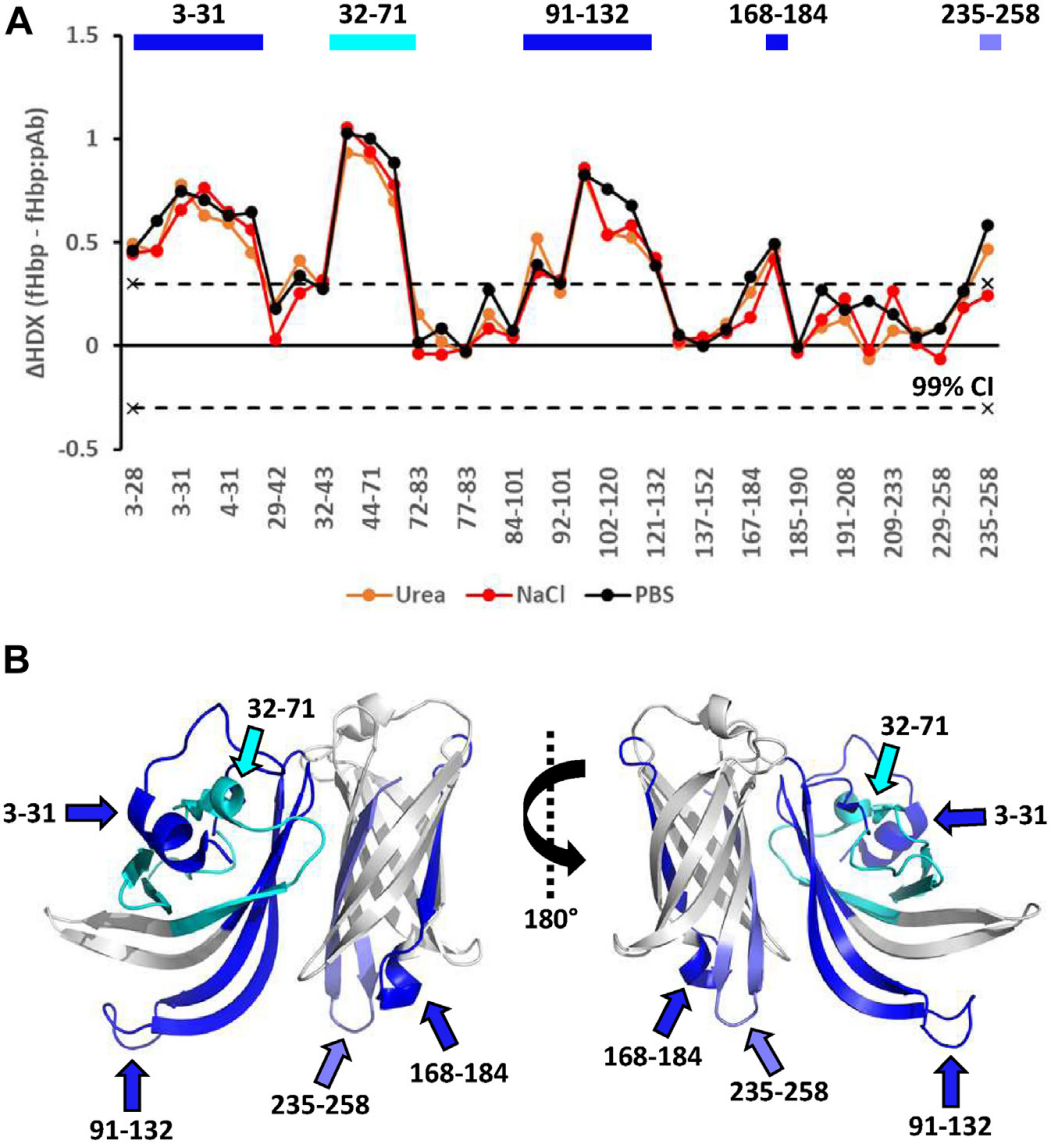
**Epitope mapping of fHbp with pAb from HD2**. *A*, the epitope mapping was performed under regular conditions (*black*), or in the presence of 0.5 M urea (*orange*) or 0.5 M NaCl (*red*). The ΔHDX is depicted on the y-axis and the identified peptides are listed on the x-axis starting from the N-terminus of fHbp. Positive and negative ΔHDX values in the presence of pAb indicate reduced and induced HDX, respectively. The dotted lines indicate the 99% CI (ΔHDX ±0.3 D). *B*, the peptides showing significant reduction in HDX in presence of pAb are illustrated on the crystal structure of fHbp (PDB 3KVD) using PyMOL.

Having established that the pAb from HD1 contained enough fHbp-specific Ab for detecting immunodominant epitopes, we extracted the pAb from a second donor, HD2. Epitope mapping was performed in the absence and presence of either 0.5 M urea or 0.5 M NaCl to avoid unspecific binding as described previously (20). The 10-minute timepoint was used for triplicate measurements. A total of 37 peptides were identified, corresponding to a sequence coverage of 99% with a mean back-exchange of 33%. Generally, the protection from HDX in the presence of pAb and in the absence and presence of urea or salt did not show any significant differences (Fig. 3). In the presence of pAb, significant reduction in HDX was observed for all conditions in residues 3 to 71 and 91 to 132, while residues 168 to 184 and 235 to 258 showed smaller yet significant reductions. In conclusion, the immunodominant epitopes of fHbp recognized by the pAb from HD2, localize to residues 3 to 71 and 91 to 132, while residues 168 to 184 and 235 to 258 may comprise less dominant epitopes or be the result of allosteric effects upon binding.

### Epitope Mapping of fHbp with Purified fHbp-specific pAb from HD2

**Fig. 4:**
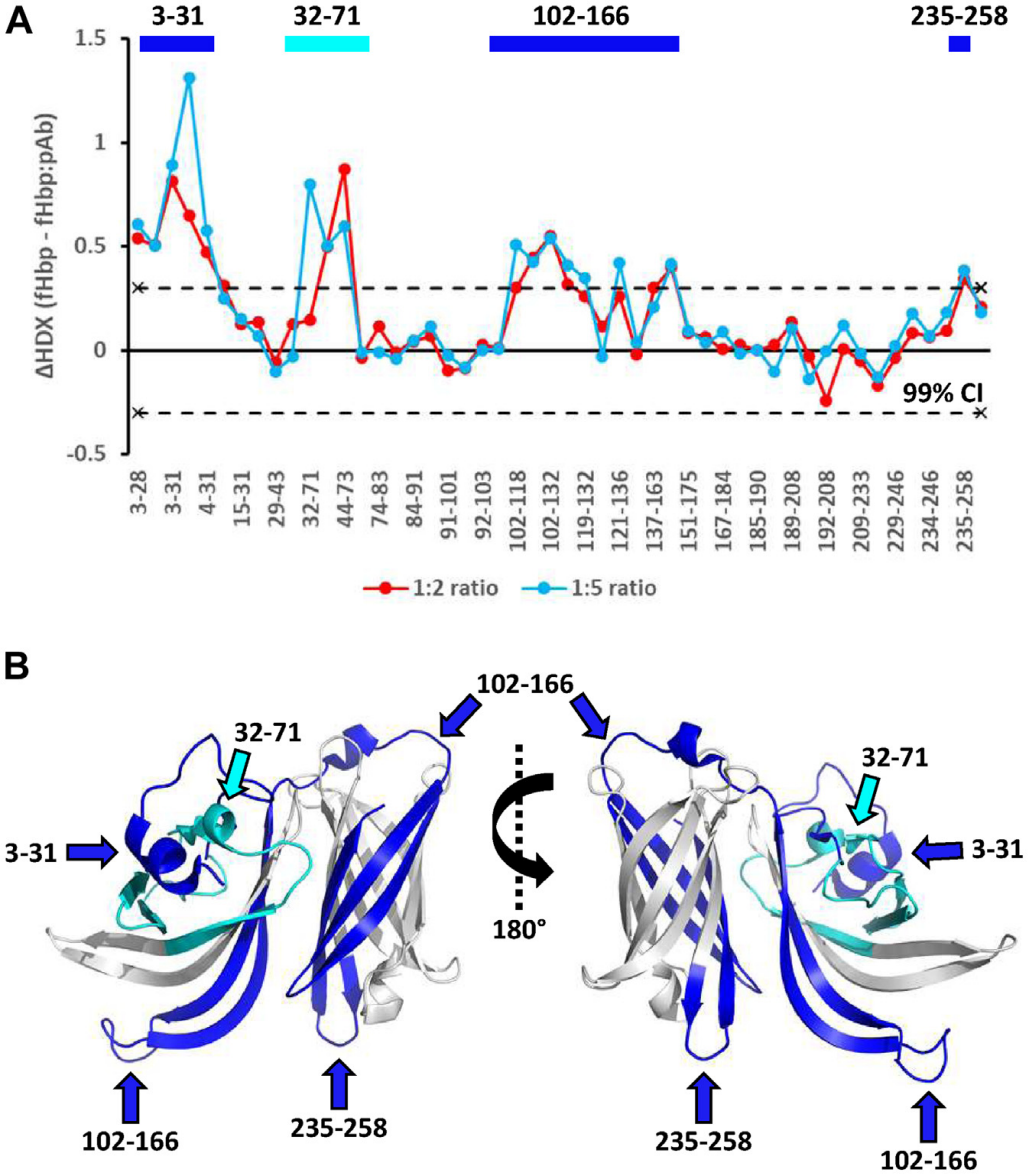
**Epitope mapping of fHbp with fHbp-specific pAb from HD2**. *A*, the epitope mapping was performed in two ratios 1:2 (*red*) and 1:5 (*blue*). The ΔHDX is depicted on the y-axis and the identified peptides are listed on the x-axis starting from the N-terminus of fHbp. Positive and negative ΔHDX values in the presence of pAb indicate reduced and induced HDX, respectively. The *dotted lines* indicate the 99% CI (ΔHDX ±0.3 D). *B*, the peptides showing significant reduction in HDX in presence of pAb are illustrated on the crystal structure of fHbp (PDB 3KVD) using PyMOL.

To further investigate impact of pAb sample complexity on the binding epitopes on fHbp, we affinity purified the pAb from HD2 using a column with immobilized fHbp to obtain a pAb with only fHbp-specific Abs. Two ratios (1:2 and 1:5) of the fHbp-specific pAb were used for epitope mapping of fHbp. Both the 10 and 100 min time points were used for triplicate measurements. A total of 50 peptides were identified, corresponding to a sequence coverage of 99%. In the presence of fHbp-specific pAb, significant protection from HDX was observed in residues 3 to 15, 32 to 71 and 102 to 166, while residues 235 to 258 showed smaller yet significant protection (Fig. 4).

The identified epitopes comprising residues 3 to 15, 32 to 71 and the less dominant epitope comprising residues 235 to 258 are in good agreement with the epitope mapping data of the total pAb population. The epitope comprising residues 102 to 166 recognized by the fHbp-specific pAb overlap with the epitope comprising residues 91 to 132 recognized by the pAb. Interestingly, the epitope comprising residues 15 to 31 and 168 to 184 recognized by the pAb was not recognized by the fHbp-specific pAb, which could indicate that these are in fact not part of the immunodominant epitopes and that the Abs recognizing these two epitopes are smaller subpopulation that was lost during further purification likely due to lower binding affinity. Another explanation could simply be that the 1:2 and 1:5 ratios used with the fHbp-specific pAb are too low to detect all epitopes. Finally, we note that in an HDX-MS epitope mapping experiment using pAb samples, there could be subpopulations of fHbp with different antibodies bound to different epitopes. This could result in broadened isotopic envelopes for peptides spanning the regions of fHbp that display changes in HDX upon addition of pAb. However, we did not detect such phenomenon. One explanation could be that the modest magnitude of the differences in HDX observed by the presence of pAb (<1 Da) limits detection of a broadening of the isotopic envelope due to any heterogeneity in the binding effect. Another explanation could be that the identified epitopes are immunodominant and recognized by the most abundant antibodies in the pAb.

## Discussion

Here we show that the complex mixture of human pAb from vaccinated individuals can be directly used for mapping of the immunodominant epitopes on a protein antigen by HDX-MS.

From the epitope mapping of fHbp with pAb from HD1 and HD2, it is clear that residues 3 to 15, 44 to 71 and 102 to 118 are the shortest common immunodominant epitopes recognized by pAbs from both donors. Less dominant epitopes comprising residues 15 to 32 and 235 to 246 were also identified in both donors. Epitope mapping of fHbp-specific pAb from HD2 revealed immunodominant epitopes that overlapped with those identified for the total pAb population from the same HD, indicating that epitope mapping with a pAb sample does not necessarily require purification of the antigen-specific Ab. The main difference observed in the epitope mapping of total pAb and fHbp-specific pAb from HD2 was the epitope comprising residues 168 to 184, which was identified in the total pAb sample but not in the fHbp-specific sample. Two mAbs produced from the B-cell repertoire of HD1 have previously been used for epitope mapping with HDX-MS, and the epitopes that they recognize overlap with the epitopes that we identified for the pAb from HD1. This indicates that HDX-MS epitope mapping with a pAb sample can allow simultaneous identification of epitopes recognized by different mAbs. We conclude that the use of HDX-MS for epitope mapping with total pAb samples could be an advantageous approach for fast screening of immunodominant epitopes in all stages of vaccine development and in studies of natural immunity against new pathogens.

## Data Availability

The HDX-MS data (including HDX Summary and HDX Data Tables) are available *via* ProteomeXchange with the identifier PXD046572 and PXD047712.

## Supplemental data

This article contains supplemental data.

## Conflict of interest

The authors declare the following financial interests/personal relationships which may be considered as potential competing interests: Nathalie Norais is a permanent employee of the GSK group of companies. Laura R. Grauslund and Susanne Ständer were employees of the GSK group of companies and PhD students at the University of Copenhagen at the time of this study. The work was funded by the 10.13039/501100007601Horizon 2020 EU framework program for research and innovation (VADEMA grant agreement no. 675879) and the 10.13039/501100000781European Research Council (ERC) (vAMRes grant agreement no. 787552). Bexsero is a trademark owned by or licensed to the GSK group of companies.
