# Supplementary material for: Epitope Mapping of Human Polyclonal Antibodies to the fHbp Antigen of a Neisseria Meningitidis Vaccine by Hydrogen-Deuterium Exchange Mass Spectrometry (HDX-MS)
Source: Mol Cell Proteomics. 2024 Feb 9;23(3):100734. doi: 10.1016/j.mcpro.2024.100734 (PMC10959699; doi:10.1016/j.mcpro.2024.100734)

**SUPPLEMENTARY FIGURES**

**Figure S1.** Coverage map of fHbp from pAb ratio test (HD1).


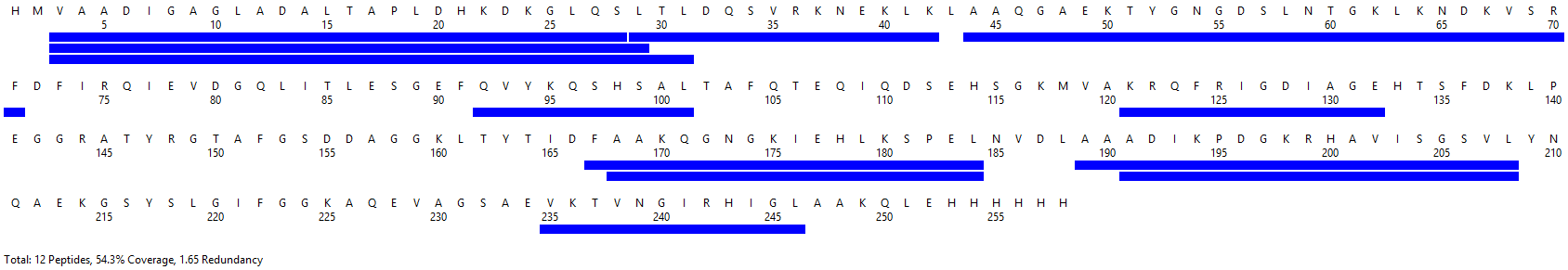


**Figure S2.** Uptake plots from ratio test (HD1).

fHbp 1:2 ratio

fHbp 1:6 ratio

fHbp 1:12 ratio

fHbp 1:2 ratio

fHbp 1:6 ratio

fHbp 1:12 ratio

fHbp 1:2 ratio

fHbp 1:6 ratio

fHbp 1:12 ratio

fHbp 1:2 ratio

fHbp 1:6 ratio

fHbp 1:12 ratio

**Figure S3.** Difference plot of fHbp with and without 0.5M urea. **
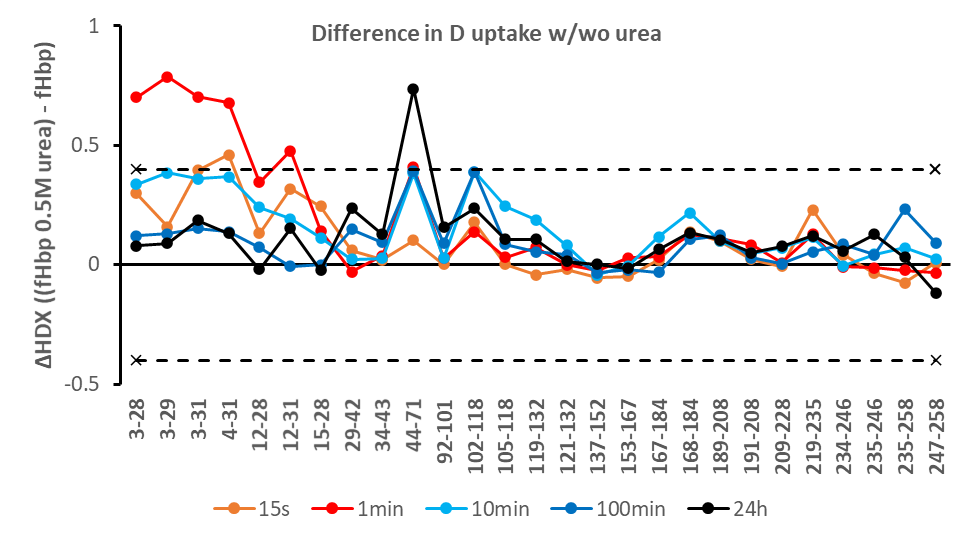
**

**Figure S4.** Coverage map of fHbp from epitope mapping with pAb (HD1).


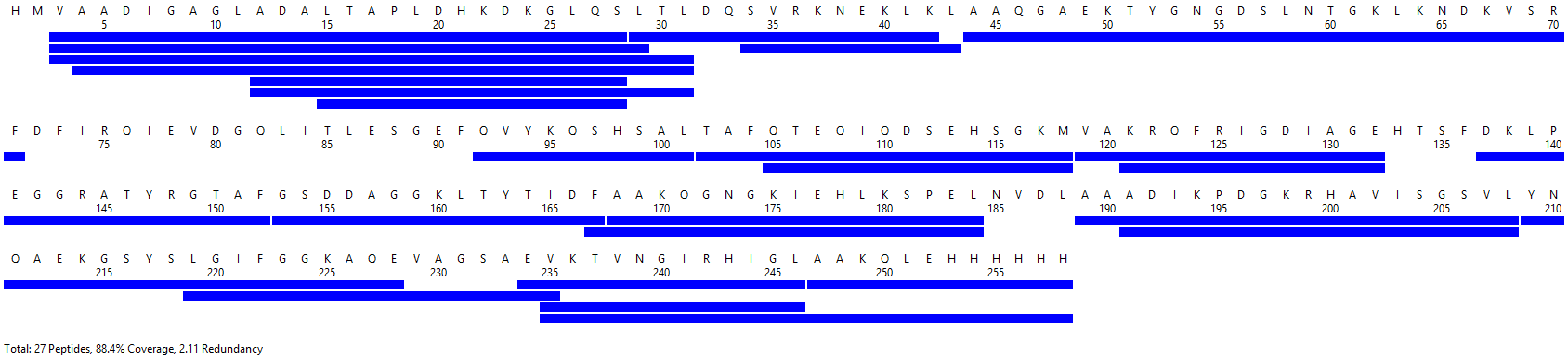


**Figure S5.** Uptake plots from epitope mapping of fHbp with pAb (HD1).

fHbp:pAb (HD1)

fHbp:pAb 0.5M urea (HD1)

fHbp:pAb (HD1)

fHbp:pAb 0.5M urea (HD1)

fHbp:pAb (HD1)

fHbp:pAb 0.5M urea (HD1)

fHbp:pAb (HD1)

fHbp:pAb 0.5M urea (HD1)

fHbp:pAb (HD1)

fHbp:pAb 0.5M urea (HD1)

fHbp:pAb (HD1)

fHbp:pAb 0.5M urea (HD1)

fHbp:pAb (HD1)

fHbp:pAb 0.5M urea (HD1)

**Figure S6.** Coverage map of fHbp from epitope mapping with total pAb (HD2).

**Figure S7.** Uptake plots from epitope mapping with total pAb (HD2).

fHbp:pAb (HD2)

fHbp:pAb 0.5M urea (HD2)

fHbp:pAb 0.5M NaCl (HD2)

fHbp:pAb (HD2)

fHbp:pAb 0.5M urea (HD2)

fHbp:pAb 0.5M NaCl (HD2)

fHbp:pAb (HD2)

fHbp:pAb 0.5M urea (HD2)

fHbp:pAb 0.5M NaCl (HD2)

fHbp:pAb (HD2)

fHbp:pAb 0.5M urea (HD2)

fHbp:pAb 0.5M NaCl (HD2)

fHbp:pAb (HD2)

fHbp:pAb 0.5M urea (HD2)

fHbp:pAb 0.5M NaCl (HD2)

fHbp:pAb (HD2)

fHbp:pAb 0.5M urea (HD2)

fHbp:pAb 0.5M NaCl (HD2)

fHbp:pAb (HD2)

fHbp:pAb 0.5M urea (HD2)

fHbp:pAb 0.5M NaCl (HD2)

fHbp:pAb (HD2)

fHbp:pAb 0.5M urea (HD2)

fHbp:pAb 0.5M NaCl (HD2)

fHbp:pAb (HD2)

fHbp:pAb 0.5M urea (HD2)

fHbp:pAb 0.5M NaCl (HD2)

fHbp:pAb (HD2)

fHbp:pAb 0.5M urea (HD2)

fHbp:pAb 0.5M NaCl (HD2)

**Figure S8.** Coverage map of fHbp from epitope mapping with fHbp-specific pAb (HD2).

**Figure S9.** Uptake plots from epitope mapping with fHbp-specific pAb (HD2).

fHbp:pAb fHbp-specific 1:2 ratio (HD2)

fHbp:pAb fHbp-specific 1:5 ratio (HD2)

fHbp:pAb fHbp-specific 1:2 ratio (HD2)

fHbp:pAb fHbp-specific 1:5 ratio (HD2)

fHbp:pAb fHbp-specific 1:2 ratio (HD2)

fHbp:pAb fHbp-specific 1:5 ratio (HD2)

fHbp:pAb fHbp-specific 1:2 ratio (HD2)

fHbp:pAb fHbp-specific 1:5 ratio (HD2)

fHbp:pAb fHbp-specific 1:2 ratio (HD2)

fHbp:pAb fHbp-specific 1:5 ratio (HD2)

fHbp:pAb fHbp-specific 1:2 ratio (HD2)

fHbp:pAb fHbp-specific 1:5 ratio (HD2)

fHbp:pAb fHbp-specific 1:2 ratio (HD2)

fHbp:pAb fHbp-specific 1:5 ratio (HD2)

fHbp:pAb fHbp-specific 1:2 ratio (HD2)

fHbp:pAb fHbp-specific 1:5 ratio (HD2)

fHbp:pAb fHbp-specific 1:2 ratio (HD2)

fHbp:pAb fHbp-specific 1:5 ratio (HD2)


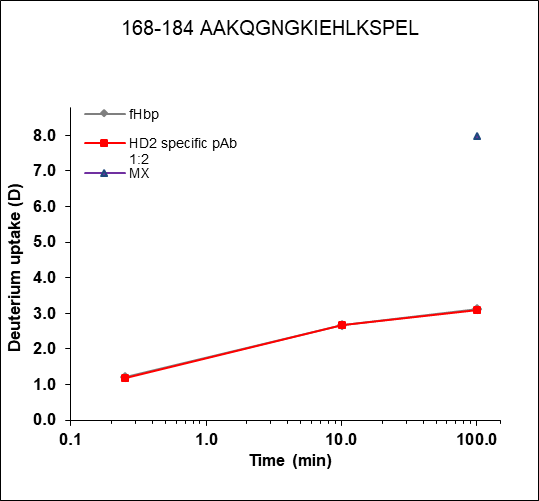

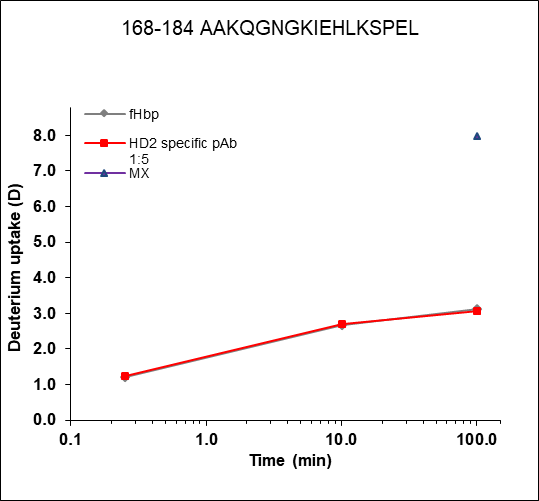


fHbp:pAb fHbp-specific 1:2 ratio (HD2)

fHbp:pAb fHbp-specific 1:5 ratio (HD2)


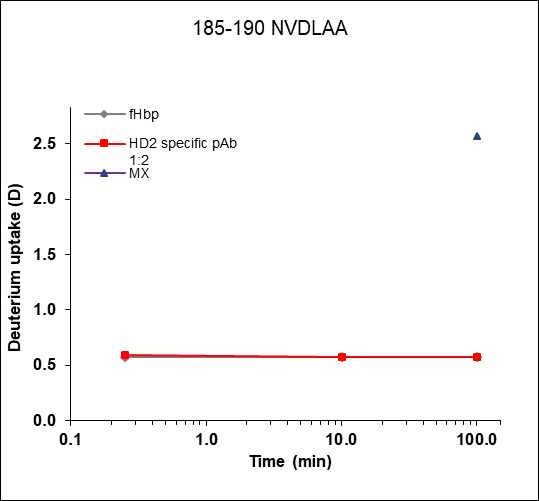

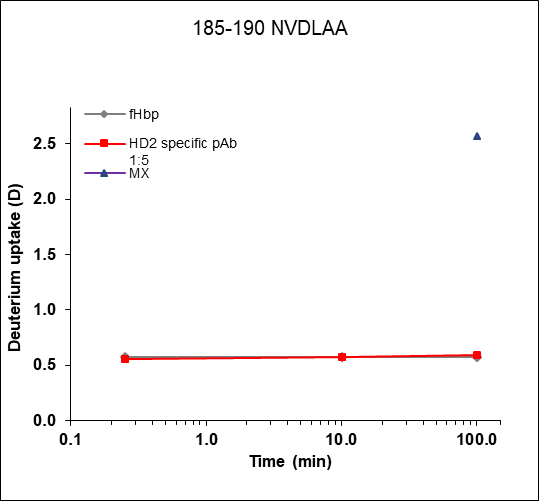


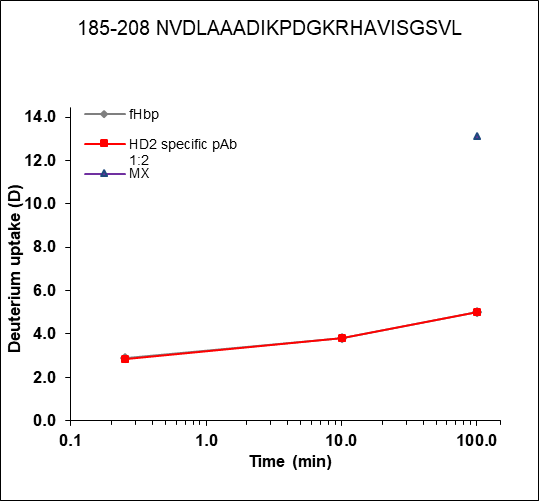

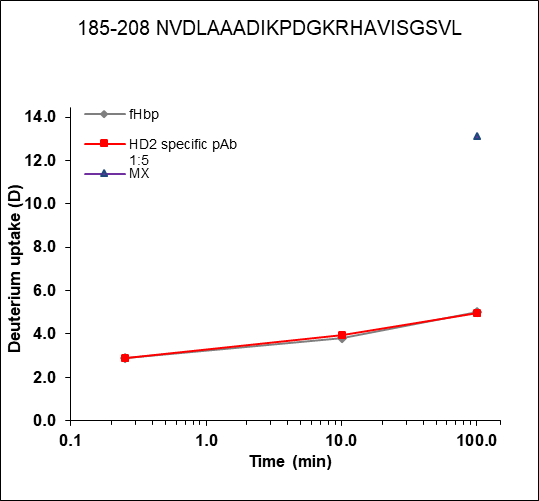


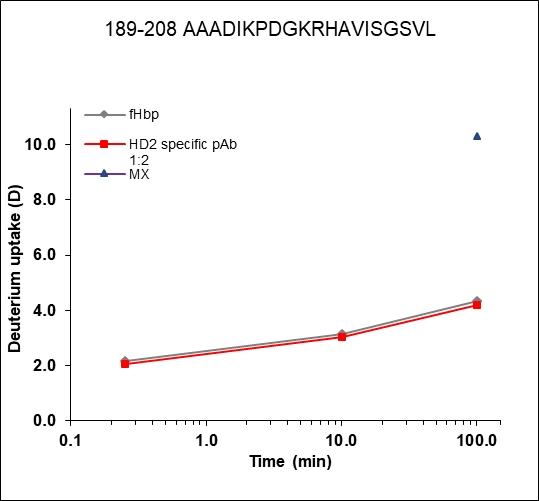

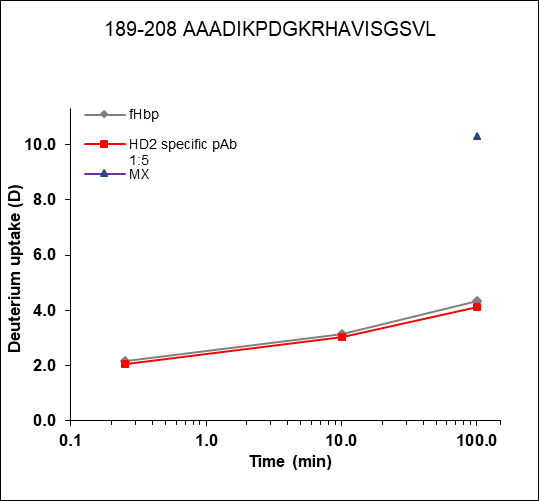


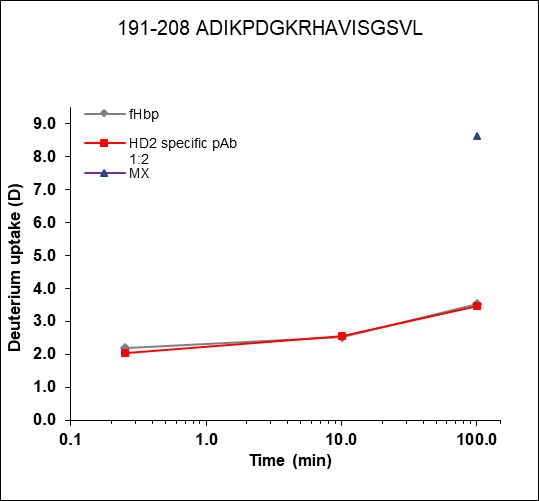

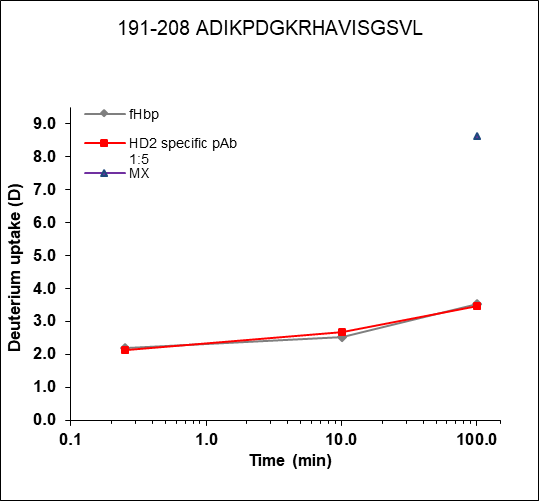


fHbp:pAb fHbp-specific 1:2 ratio (HD2)

fHbp:pAb fHbp-specific 1:5 ratio (HD2)


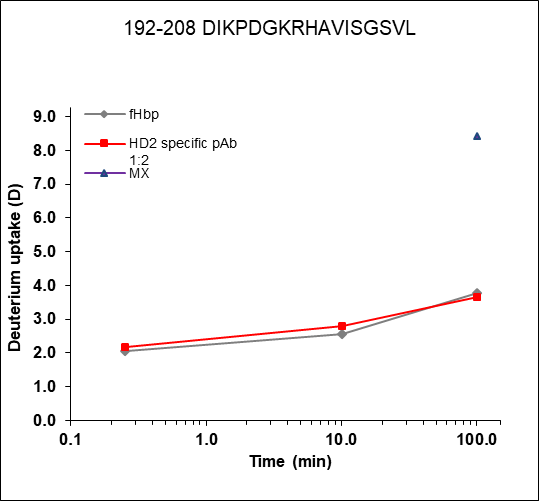

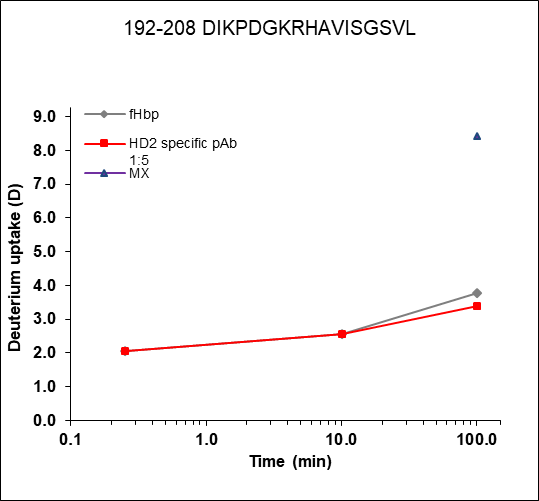


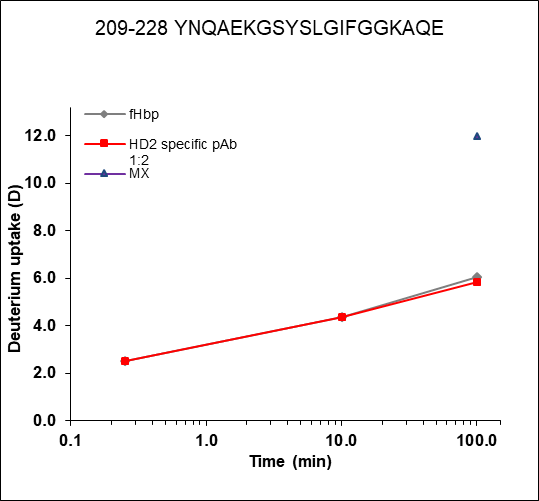

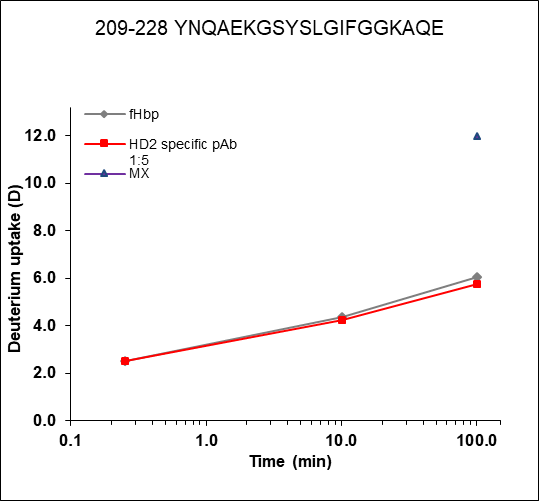


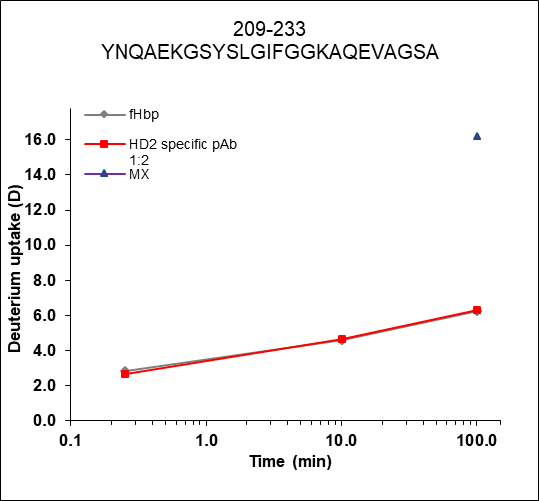

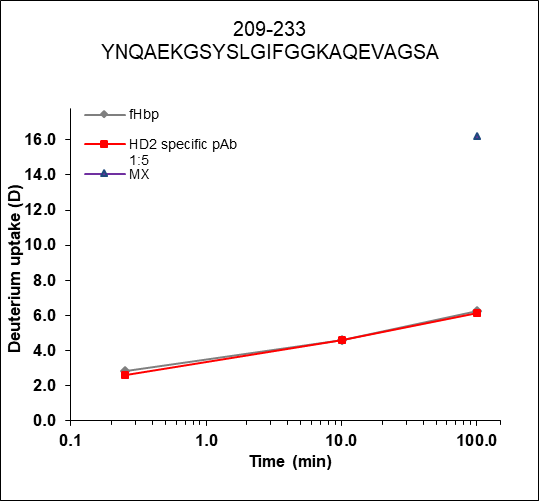


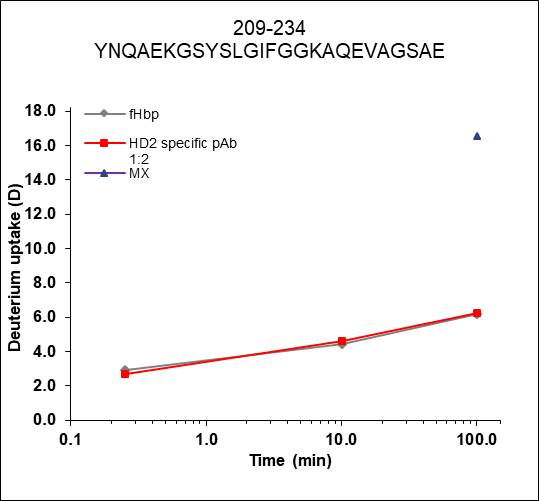

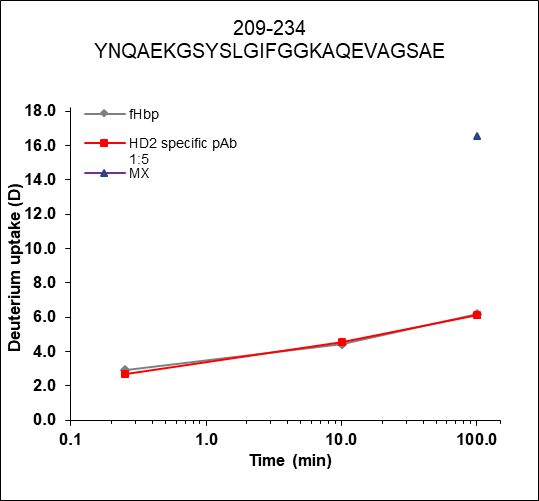


fHbp:pAb fHbp-specific 1:2 ratio (HD2)

fHbp:pAb fHbp-specific 1:5 ratio (HD2)


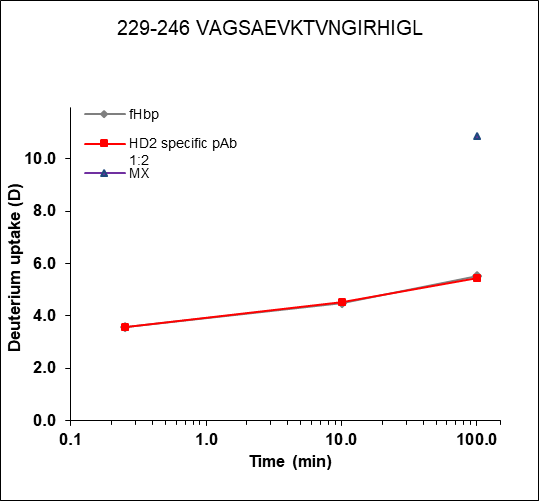

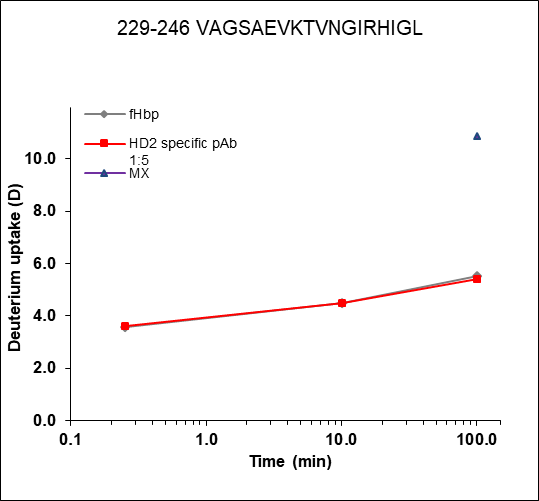


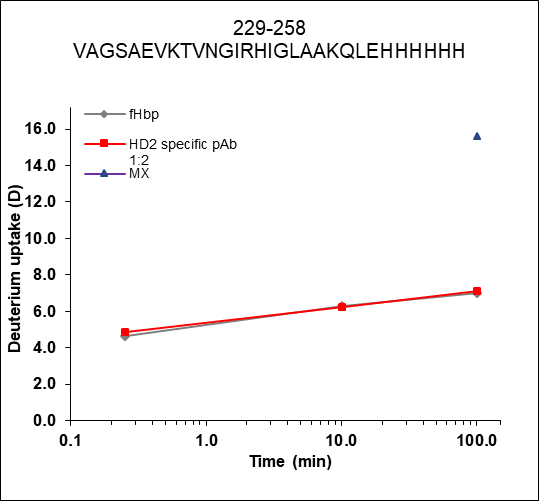

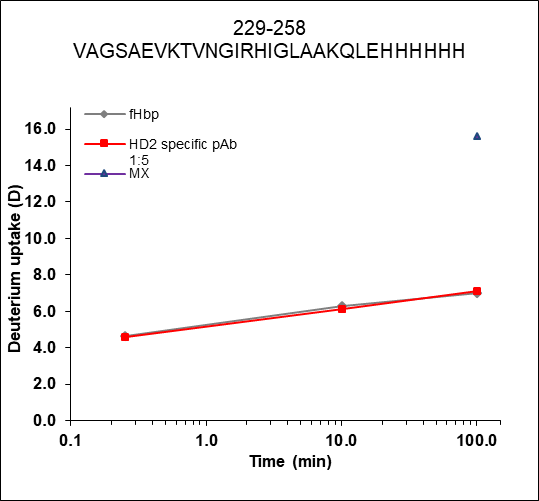


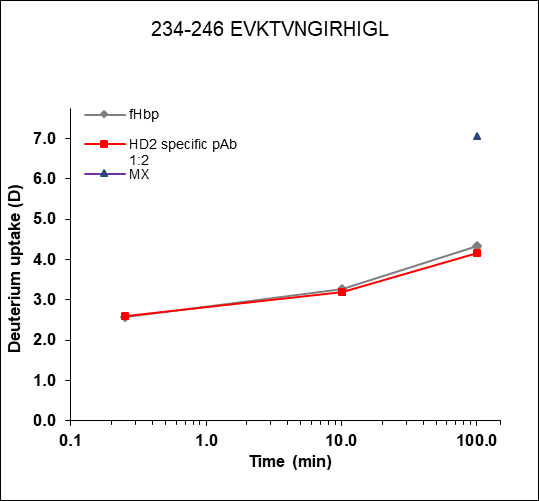

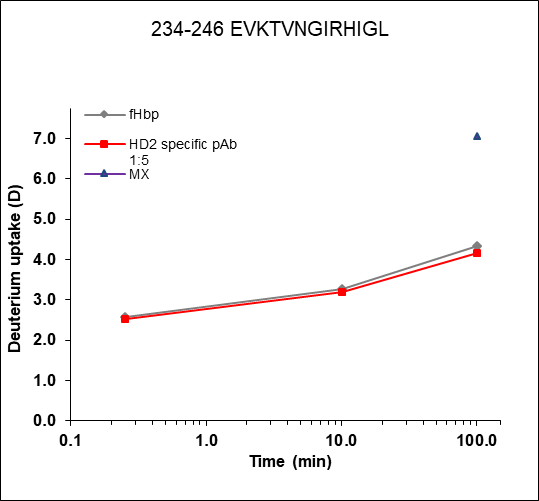


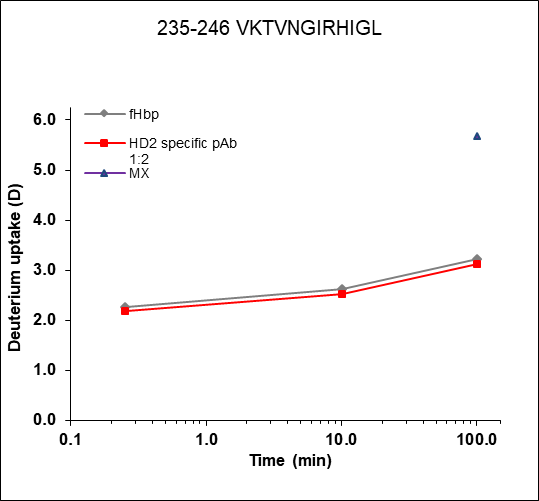

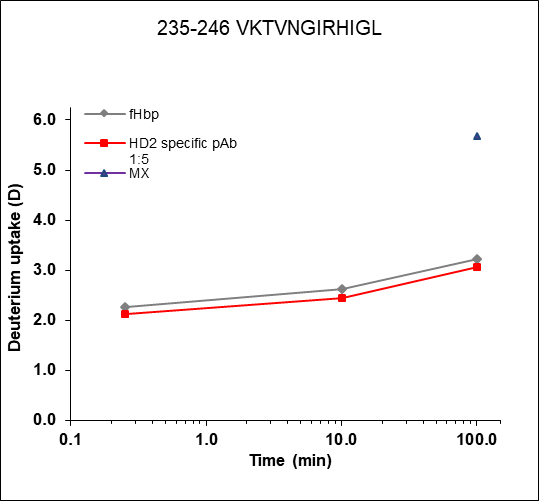


fHbp:pAb fHbp-specific 1:2 ratio (HD2)

fHbp:pAb fHbp-specific 1:5 ratio (HD2)


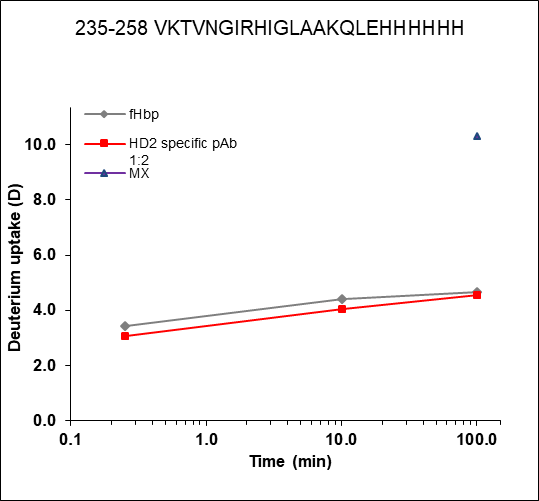

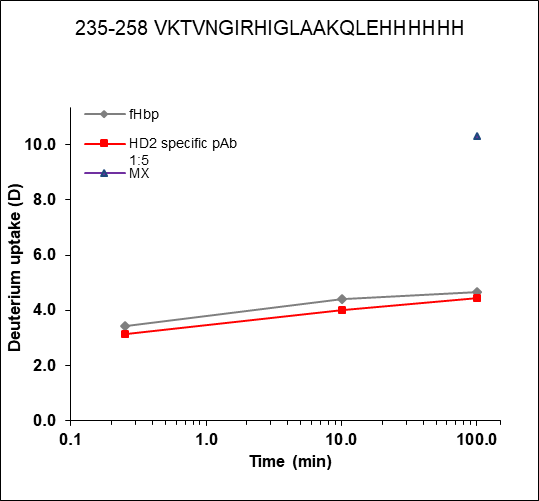


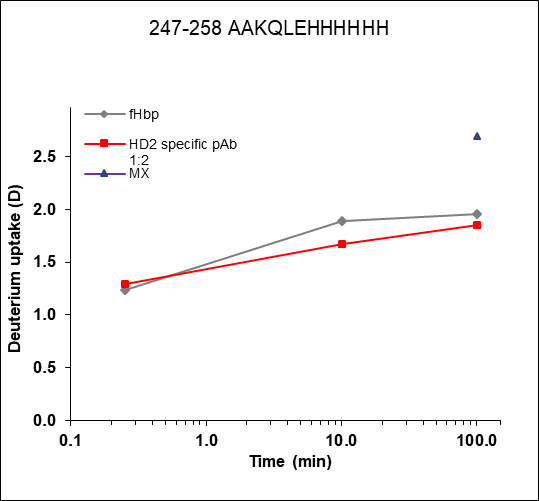

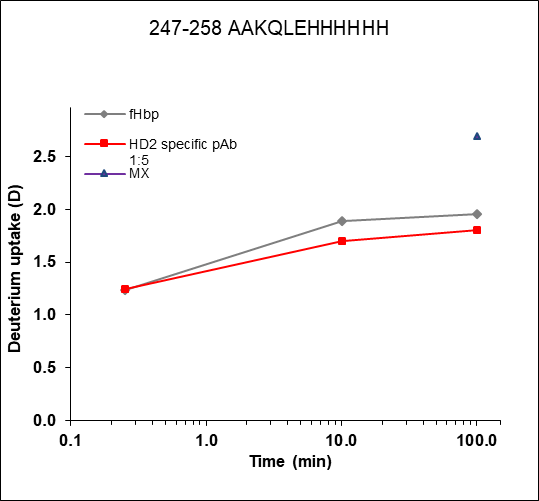

Supplement: Supplementary Figures [file mmc1.docx]
